# Exploring phylogeny and genomic vulnerability of *Melastoma* (Melastomataceae) endemic to a World Natural Heritage site, the Bonin Islands

**DOI:** 10.1038/s41598-024-65726-6

**Published:** 2024-07-17

**Authors:** Yukihiro Kobayashi, Yoshiteru Komaki, Yuji Isagi

**Affiliations:** 1 Graduate School of Agriculture, Kyoto University, Kyoto, Japan; 2 Botanical Gardens, Graduate School of Science, The University of Tokyo, Tokyo, Japan

**Keywords:** Phylogeny, Endemic species, Endangered species, Conservation genomics, Ecology, Biodiversity, Conservation biology, Ecological genetics

## Abstract

*Melastoma* (Melastomataceae) includes ca. 100 species across tropical to subtropical regions of Asia and Oceania. The Bonin Islands harbor three endemic taxa: *M. tetramerum*, *M. tetramerum* var. *pentapetalum*, and *M. candidum* var. *alessandrense*. Of these, *M. tetramerum* is critically endangered and faces near extinction in the wild. This study investigates the phylogenetic relationships among these endemic *Melastoma* species in the Bonin Islands based on the whole chloroplast genome and nuclear SNPs. The results revealed that *M. candidum* var. *alessandrense* was placed in the clade of the widespread East Asian *M. candidum* and has a distinct evolutionary origin from the other two taxa. The population genomics analyses (heterozygosity, rates of deleterious mutations, and numbers and lengths of runs of homozygosity) indicated lower genetic diversity and more vulnerable genomes of endemic *Melastoma*, especially *M. tetramerum* var. *pentapetalum*. *M. tetramerum* var. *pentapetalum* is not a target of any protection programs, however, conservation plans might be required for this variety because *M. tetramerum* var. *pentapetalum* would have a more vulnerable genome than *M. tetramerum,* which faces near extinction in the wild. This information can facilitate the development of effective conservation strategies in a precautionary way that anticipates imminent threats to the survival of the species.

## Introduction

Situated approximately 1000 km south of Tokyo in the Pacific Ocean, the Bonin Islands represent a typical oceanic archipelago. Notably, these islands have never been connected to the mainland since its formation. This isolation has fostered distinct evolutionary pathways, separate from those observed on the continent and mainland Japan. Consequently, the Bonin Islands boast a unique and remarkable ecosystem. The islands serve as a repository for invaluable evidence of evolutionary processes. This includes adaptive radiation, for example, in land snails and vascular plants. Despite their modest size, the Bonin Islands exhibit an exceptionally high level of endemism, and this remarkable biodiversity ultimately led to their designation as a UNESCO World Natural Heritage Site in 2011. However, a significant number of taxa endemic to the Bonin Islands are facing the threat of extinction. Therefore, the prompt implementation of effective conservation measures is of paramount importance.

*Melastoma* L. is a genus of Melastomataceae and consists of approximately 100 species in tropical to subtropical Asia and Oceania^1^. Many *Melastoma* species are widely cultivated in tropical to subtropical regions for medicinal or horticultural purposes^2^. The Bonin Islands harbor three endemic *Melastoma* taxa, all of which are listed in the Red List of the Ministry of the Environment: *M. candidum* D. Don var. *alessandrense* S.Kobay. classified as vulnerable (VU), *M. tetramerum* Hayata as critically endangered (CR), and *M. tetramerum* var. *pentapetalum* Toyoda as endangered (EN). The number of individuals of the three rare taxa that remained in wild habitats is less than 100 for *M. tetramerum* and *M. tetramerum var. Pentapetalum* (personal observation by Yoshiteru Komaki) and about 1000 for *M. Candidum var. Alessandrese* (personal observation by Dairo Kawaguchi). Among these endemic taxa, M. tetramerum is nearly extinct in wild habitats and is listed as a target species for the protection and reproduction programs of the Ministry of the Environment, Japan. 64 individuals of *M. tetramerum* have been artificially propagated and reintroduced to wild habitats. The Bonin Islands, isolated from nearest landmasses, harbor plant species endemic to the archipelago, making them important for investigating plant evolution, particularly adaptive radiations and long-distance dispersals. The plant species endemic to the Bonin Islands have diverse geographical origins, with some tracing back to the Okinawa Islands or southeast Asia^3^ and others to the Pacific islands^4,5^. The origin of the *Melastoma* taxa endemic to the Bonin Islands, however, remains unclear due to limitations in the resolution of previous phylogenetic trees^6^.

Generally, endangered species have more vulnerable genomes than common species in terms of genetic diversity (e.g. heterozygosity), rates of duplicated genes, and rates of deleterious mutations due to inbreeding in small populations^7^. Low diversity and deleterious mutations could lead to low fertility of seeds or high vulnerability to changing environments or introduction of diseases. On the contrary, several endangered species with small population sizes have lower rates of deleterious mutations^8^, suggesting that extinction risk can be different among endangered species. This highlights the importance of evaluating genomic vulnerability within endangered species, especially for effective conservation planning. Among the endemic *Melastoma* taxa of the Bonin Islands, the genome of *M. tetramerum* is shown to have lower genetic diversity and a higher rate of deleterious mutations^7^. However, the genomic vulnerability of *M. candidum* var. *alessandrense* and *M. tetramerum* var. *pentapetalum* remains unknown. To develop effective conservation plans, the evaluation of genomic vulnerability is quite important because loss of genetic diversity sometimes causes the breakdown of populations even if conservation programs are conducted by ex situ propagation^9^.

This study employed molecular phylogenetic analyses using whole nuclear genome SNPs and chloroplast sequences to investigate the origins of *Melastoma* endemic to the geographically isolated Bonin Islands. Population genomics analyses (estimation of heterozygosity, numbers of multi-allelic sites, rates of deleterious mutations, and length of runs of homozygosity) and demographic inference were also performed to assess their genomic vulnerability to inform effective conservation plans.

## Results

### Number of reads and mapping rates

After adapter removal and quality filtering, short read sequencing yielded a total of 469,211,051 reads from 23 individuals. The range of the number of reads per sample was 5,109,066–65,263,874 reads, with an average of 20,400,480 reads. The range of mapping rates to the reference nuclear genome sequence was 22.55% to 90.04%, with an average of 68.56%.

### Phylogenetic analysis based on the whole genome of chloroplast

**Figure 1:**
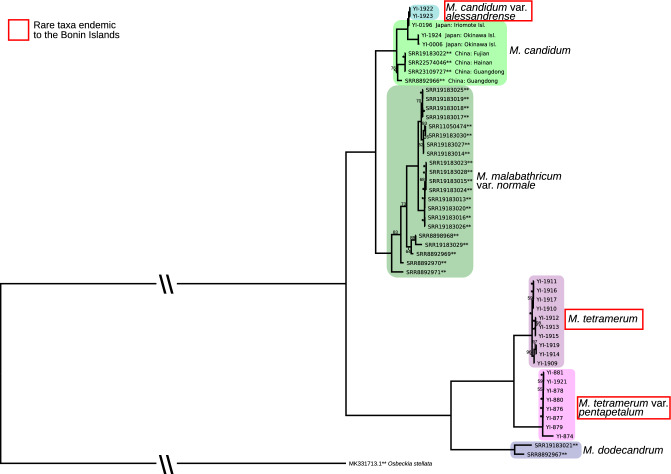
The maximum likelihood tree based on the whole chloroplast sequences (139,334 bp after alignment) with the GTR + G + I model. The log likelihood value was − 201192.1681. The numbers on branches were bootstrap values from 100 replications. The bootstrap values of the branches without any number were 100, and asterisks represented bootstrap values lower than 50. **: The samples sourced from NCBI GenBank.

After alignment, removing one copy of the inverted repeat region, and trimming, a sequence length of chloroplast genome of 139,334 bp was used for phylogenetic analysis. The maximum likelihood tree based on chloroplast sequences revealed two major clades with 100% bootstrap values (BS) (Fig. 1). One clade included *Melastoma candidum*, *M. candidum* var. *alessandrense*, and *M. malabathricum* var. *normale*. *Melastoma candidum* var. *alessandrense* was nested within the individuals of *M. candidum* sampled from the Okinawa Islands. The other included *M. dodecandrum*, *M. tetramerum*, and *M. tetramerum* var. *pentapetalum*. All the endangered taxa native to the Bonin Islands were monophyletic with 100% BS, respectively.

### Phylogenetic analysis based on the whole nuclear genome SNPs

**Figure 2:**
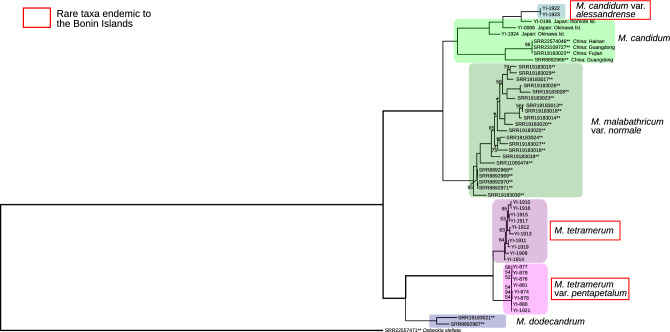
The maximum likelihood tree based on the whole genome SNPs data (3,476,070 bp). Only SNPs shared by 80% or more samples were used for the analysis. The GTR + G model was applied, and the log likelihood value was − 5889107.197. The numbers on branches were bootstrap values from 100 replications. The bootstrap values of the branches without any number were 100, and asterisks represented bootstrap values lower than 50. **: The samples sourced from NCBI GenBank.

The maximum likelihood phylogenetic tree was constructed based on 3,476,070 SNPs. The topology of the SNPs tree was nearly identical to the chloroplast tree, i.e., one clade including *Melastoma candidum*, *M. candidum* var. *alessandrense* (nested within *M. candidum* from the Okinawa Islands), and *M. malabathricum* var. *normale*, and the other including *M. dodecandrum*, *M. tetramerum*, and *M. tetramerum* var. *pentapetalum* (Fig. 2). Notably, the branch length of the endangered taxa native to the Bonin Islands were shorter than other species.

### Demographic inference

**Figure 3:**
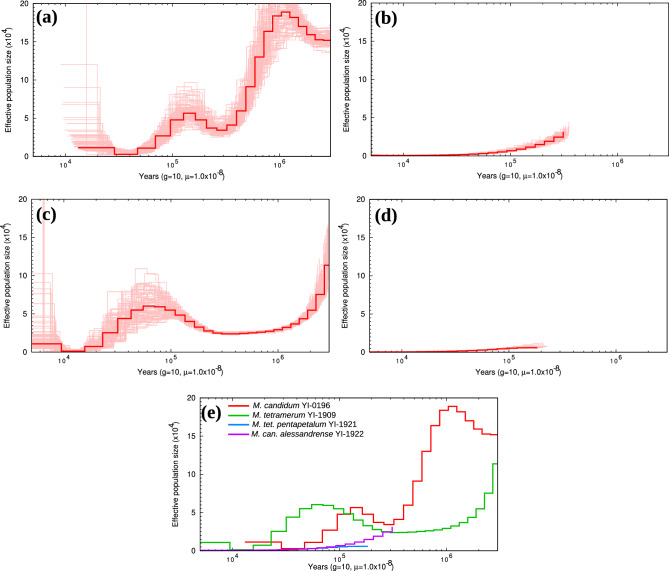
The ancient population dynamics of four *Melastoma* taxa estimated by PSMC analyses. (**a**) *M. candidum*, (**b**) *M. candidum* var. *alessandrense*, (**c**) *M. tetramerum*, (**d**) *M. tetramerum* var. *pentapetalum*, and (**e**) All taxa. Thin lines in (**a**), (**b**), (**c**), and (**d**) showed the results of 100 bootstrap replications. Abbreviations;* M*. *tet. pentapetalum*: *M. tetramerum* var. *pentapetalum*, *M*. can. *alessandrense*: *M. candidum* var. *alessandrense.*

The results of demographic inference by PSMC were shown in Fig. 3. The reliable range was different in each sample based on the results of 100 bootstrap replications: The analysis revealed that the reliability range of estimated effective population size varies among samples. *Melastoma candidum* (YI-0196) would be reliable between approximately 20,000–2,500,000 years, *M. candidum* var. *alessandrense* (YI-1923) 5000–200,000 years, *M. tetramerum* (YI-1909) 9000–2,500,000 years, and *M. tetramerum* var. *pentapetalum* (YI-1921) 5000–200,000 years. Notably, *M. candidum* and *M. tetramerum* exhibited increases of population sizes around 100,000 years ago. Conversely, the population sizes of *M. candidum* var. *alessandrense* and *M. tetramerum* var. *pentapetalum* have shown a continuous decline.

### Population genomics analyses and statistics

**Figure 4:**
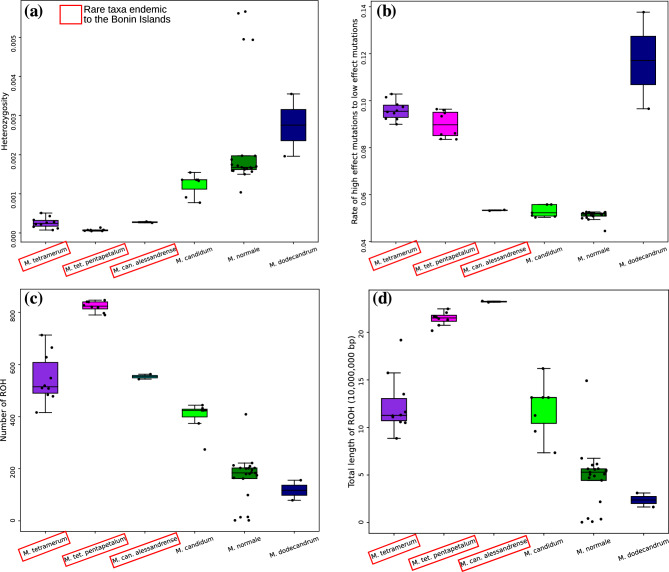
The scattered boxplot of the results of analyses on the genetic diversity of six *Melastoma* taxa. (**a**) Heterozygosity, (**b**) rate of high-effect mutations to low-effect mutations, (**c**) number of runs of homozygosity on chromosome 1–12, and (**d**) total length of runs of homozygosity on chromosome 1–12. The ROH regions only longer than 100,000 bp were used for the analyses. Abbreviations;* M*. *tet. pentapetalum*: *M. tetramerum* var. *pentapetalum*, *M*. can. *alessandrense*: *M. candidum* var. *alessandrense, M. normale*: *M. malabathricum* var. *normale*.

The heterozygosity of the three endemic taxa was lower than common species (*M. candidum*) and the difference between *M. tetramerum* and *M. candidum* (*p*-value (*p*) was 0.00199), and *M. tetramerum* var. *pentapetalum* and *M. candidum* (*p* = 0.00404) were significant (Fig. 4a, Table 1). The heterozygosity of *M. tetramerum* var. *pentapetalum* was lower than *M. tetramerum* (*p* = 4.57e − 5). The rate of high-effect mutations of the three endemic taxa were higher than *M. candidum* and *M. tetramerum* (*p* = 0.000103) and *M. tetramerum* var. *pentapetalum* (*p* = 0.000311) were significant (Fig. 4b, Table 1). The numbers of ROH (runs of homozygosity) of the three endemic taxa were higher than *M. candidum,* and *M. tetramerum* (*p* = 0.00397) and *M. tetramerum* var. *pentapetalum* (*p* = 0.00145) was significant (Fig. 4c, Table 1). The total length of ROH of *M. tetramerum* was similar to *M. candidum* while the other two endemic taxa had longer ROH (Fig. 4d, Table 1).

**Table 1:** The results of Mann–Whitney U test on the genetic diversity of *Melastoma* endemic to the Bonin Islands.

	*M. tetramerum*	*M. tetramerum* var. *pentapetalum*	*M. candidum*
*M. tetramerum*			
Heterozygosity	–	4.57E-5*	1.99E-3*
Rate of high effect mutations	–	6.76E-2	1.03E-4*
Number of ROH	–	4.57E-5*	3.97E-3*
Length of ROH	–	4.57E-5*	9.62E-1
*M. tetramerum* var. *pentapetalum*			
Heterozygosity	–	–	4.04E-3*
Rate of high effect mutations	–	–	3.11E-4*
Number of ROH	–	–	1.45E-3*
Length of ROH	–	–	3.11E-4*

The data for the statistical analyses were summarized in Supplementary Table S3.

*: *p*-value < 0.05.

## Discussion

There are three *Melastoma* taxa endemic to the Bonin Islands; *M. candidum* var. *alessandrense*, *M. tetramerum*, and *M. tetramerum* var. *pentapetalum*. The origins of these endemic taxa are evolutionary intriguing due to the high isolation of the Bonin Islands. Highly resolved phylogenetic analyses are essential to estimate the origins of these endemic taxa. The results of the phylogenetic analyses of this study, based on two different datasets (chloroplast sequences and whole nuclear genome SNPs), revealed nearly identical topologies (Figs. 1 and 2), suggesting two independent origins of the *Melastoma* species endemic to the Bonin Islands. *Melastoma candidum* var. *alessandrense* was included in the clade of *M. candidum* from the Okinawa Islands and the closest individual to *M. candidum* var. *alessandrense* was sampled from Iriomote island. *Melastoma tetramerum* and *M. tetramerum* var. *pentapetalum* likely have a different origin from *M. candidum* var. *alessandrense*, and these two taxa were sister to *M. dodecandrum* from China. Although *M. tetramerum* and *M. tetramerum* var. *pentapetalum* were close to *M. dodecandrum*, the branch length was quite long and there are few morphological similarities between these two endemic taxa and *M. dodecandrum*. In addition, only six taxa out of ca. 100 *Melastoma* species were analyzed in this study, then a more comprehensive analysis is necessary to accurately determine the origin of *M. tetramerum* and *M. tetramerum* var. *pentapetalum*.

**Figure 5:**
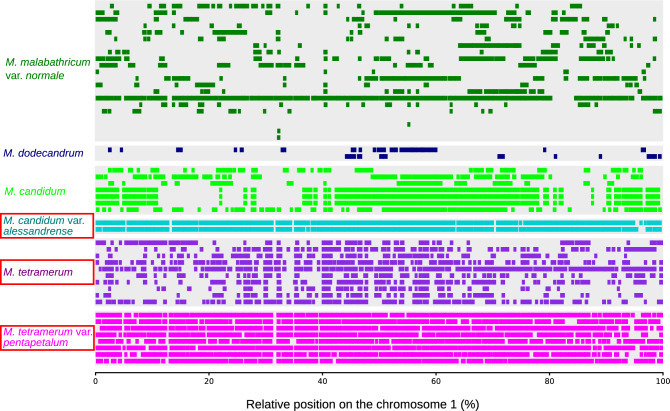
The variations of runs of homozygosity (ROH) on chromosome 1 among species and individuals. Each row represents a single individual and the ROH regions only longer than 100,000 bp were shown. Rare taxa endemic to the Bonin Islands were highlighted with red squares.

The heterozygosity, and the number and the length of ROH of *Melastoma* taxa endemic to the Bonin Islands showed that the genetic diversity of these rare taxa was lower than *M. candidum*, which is widely distributed in tropical to subtropical Asia (Figs. 4 and 5). The rate of high-effect mutations exhibited different patterns among the Bonin Island endemics. *M. tetramerum* and *M. tetramerum* var. *pentapetalum* would have more vulnerable genomes than *M. candidum*, while the genome vulnerability of *M. candidum* var. *alessandrense* was similar to *M. candidum* (Fig. 4 and Table 1). These results suggested that *M. tetramerum* and *M. tetramerum* var. *pentapetalum* are more susceptible to threats due to their genome vulnerability compared to *M. candidum* var. *alessandrense*. Rapid decreases of effective population size can sometimes cause genomic vulnerability^9^. However, *Melastoma* taxa endemic to the Bonin Islands have not experienced such rapid declines (Fig. 3). *Melastoma tetramerum* exhibited similar population dynamics to *M. candidum*, whereas the effective population sizes of *M. candidum* var. *alessandrense* and *M. tetramerum* var. *pentapetalum* have been continuously decreasing. Interestingly, despite having similar historical population dynamics, *M. candidum* var. *alessandrense* and *M. tetramerum* var. *pentapetalum* show significant differences in their genomic vulnerability. Therefore, ancient population dynamics likely do not represent the main cause factor driving the genomic vulnerability of the endemic *Melastoma* taxa.

Lower genetic diversity and higher rate of deleterious mutations were common in *M. tetramerum* and *M. tetramerum* var. *pentapetalum*. However, the extent of the genome vulnerability of these two endangered taxa were different (Figs. 4 and 5, and Table 1). In addition to heterozygosity, the number and the length of ROH were significantly different between the two taxa (*p* = 4.57e − 5). These results indicated that *M. tetramerum* var. *pentapetalum* has a more vulnerable genome compared to *M. tetramerum*. *M. tetramerum* var. *pentapetalum* comprises a larger population in its natural habitats compared to *M. tetramerum*, and is not currently designated as the focal species of the protection and reproduction programs of the Ministry of the Environment, Japan. However, considering its significance, conservation initiatives may also be necessary for this variety.

Although the multifaceted value of biodiversity is now widely recognized, many species face the threat of extinction. It is generally difficult to improve the status of species listed as endangered. Indeed, in Japan, 64 species are subject to the Conservation and Reproduction Programs under the Law for the Conservation of Species as part of biodiversity conservation, but so far no species has been de-registered as a result of improved status. This indicates that for effective conservation of species, it is imperative to implement appropriate conservation measures before the situation becomes significantly challenging. While population size provides crucial information on the conservation status of a species, genomic information can be used to implement more effective conservation measures before a species becomes endangered. Comprehensive preliminary genome analyses, such as those carried out in this study, across rare species could be crucial for long-term biodiversity conservation efforts.

## Methods

### Plant materials and DNA extraction

The collection and use of plant materials in this study were carried out in compliance with relevant institutional, national, and international guidelines and legislation. Necessary permissions and licenses were obtained from the relevant authorities (Ministry of the Environment and Forestry Agency for Japanese samples). The plant specimens were identified by two of the current authors, Yoshiteru Komaki and Yuji Isagi, and voucher specimens were deposited at Graduate School of Agriculture, Kyoto University (Supplementary Table S1). The leaves of a total of 23 individuals of *Melastoma* (three individuals of *M. candidum*: two from Okinawa Island and one from Iriomote Island, two individuals of *M. candidum* var. *alessandrense* from Kita-Iwojima Island, 10 individuals of *M. tetramerum* from Chichijima Island, and 8 individuals of *M. tetramerum* var. *pentapetalum* from Hahajima Island) were sampled and stored in silica gel. Plant materials used in this study were shown in Supplementary Table S1. DNA extraction was conducted by using the modified CTAB method^10^ from approximately 1 cm × 1 cm silica gel dried leaves.

### Library preparation, short read sequencing, and quality filtering of reads

The library was prepared with Illumina DNA Prep kit according to the manufacturer’s protocol. Sequencing was conducted by using HiSeq X Ten sequencer (Illumina, San Diego, CA, USA). The sequenced reads were preprocessed by using Trimmomatic v.0.39^11^.

### Reference genome and quoted short reads from NCBI GenBank

We used the nuclear genome of *Melastoma dodecandrum* GWHBCLA00000000^2^ as the reference sequence for the phylogenetic and population genomics analyses of this study. In addition to our original plant samples, the short read genome resequencing data of 27 individuals (four individuals of *M. candidum*, two of *M. dodecandrum*, and 21 of *M. malabathricum* var. *normale*) were downloaded from NCBI GenBank (Supplementary Table S2) and used for the following phylogenetic and population genomics analyses. All the data available on NCBI at late 2022 were used.

### Phylogenetic analysis based on the whole genome sequences of chloroplast

The whole genomes of chloroplast were assembled by using GetOrganelle v1.7.7.0^12^ with “–max-reads inf–reduce-reads-for-coverage inf” options, and the assembled sequences were aligned with MAFFT v7.249^13^ with default parameters after excluding one copy of the inverted repeat regions. After alignment, the sequence matrix was trimmed by trimAl^14^. The optimal DNA substitution model of the sequence matrix was estimated by using ModelTest-NG v0.1.7^15^. The maximum likelihood phylogenetic tree was constructed by using RAxML-NG v1.1.0^16^. The credibility of each branch was evaluated with 100 bootstrap replications. The resulting phylogenetic tree was edited by using FigTree v1.4.3^17^.

### Mapping reads to the nuclear reference genome, SNP calling, and depth filtering

The read mapping and sorting were performed by using Bowtie2 v2.5.3^18^ and SAMtools 1.19^19^. SNP calling of each sample was performed by using BCFtools 1.19^19^ with “-q 30 -Q 20” options. The average read depth (d) of each sample was calculated with SAMtools 1.19^19^, and the SNPs were filtered with the minimum threshold (d / 3; if d < 9, the minimum threshold was 3) and the maximum threshold (2 × d).

### Phylogenetic analysis based on the whole genome SNPs

The filtered vcf files were merged into a single vcf file by using the “vcf-merge” command within VCFtools v0.1.16^20^. The SNPs shared by less than 20% of individuals were discarded. The merged vcf file was converted to phylip format with vcf2phylip v2.6^21^. The maximum likelihood phylogenetic tree was constructed by using RAxML-NG v1.1.0^16^ with the GTR + G model without estimating the best DNA substitution model because of computational limitations. The credibility of each branch was evaluated with 100 bootstrap replications. The resulting phylogenetic tree was edited by using FigTree v1.4.3^17^.

### Demographic inference

The ancient population dynamics of the three taxa endemic to the Bonin Islands and *Melastoma candidum* were estimated by using PSMC^22^. For the PSMC analyses, the samples of *M. candidum* (YI-0196), *M. candidum* var. *alessandrense* (YI-1922), *M. tetramerum* (YI-1909), and *M. tetramerum* var. *pentapetalum* (YI-1921) were used. The average read depth (d) of each sample was calculated with SAMtools 1.19^19^. SNP calling was conducted by BCFtools 1.19^19^. SNPs were filtered with the minimum depth threshold (d / 3) and maximum depth threshold (2 × d). The generation time and the mutation rate were set at 10 years and 1.0e − 8, respectively.

### Population genomics analyses and statistics

To evaluate the vulnerability of the genome of the endangered *Melastoma* endemic to the Bonin Islands, the heterozygosity and the rate of high-effect mutations of each sample was estimated by using SnpEff v5.2^23^. The database of SnpEff was created from the reference genome of *M. dodecandrum*. Runs of homozygosity (ROH) was also estimated as an indicator of genetic diversity. ROH on chromosome 1–12 of each sample was estimated by using BCFtools 1.19^19^. The results were plotted with matplotlib v3.6.3^24^.

The statistical analyses were performed on the heterozygosity, the rate of high-effect mutations, and the number and the length of ROH (ROH regions longer than 100,000 bp were used for the analyses) between *M. tetramerum* and *M. candidum*, and *M. tetramerum* var. *pentapetalum* and *M. candidum* to evaluate whether the differences of the genetic diversity between these rare taxa and widely distributed species were significant. The differences between *M. tetramerum* and *M. tetramerum* var. *pentapetalum* were also analyzed. *M. candidum* var. *alessandrense* was not analyzed because there were not enough samples (only two samples) to conduct statistical analyses. We conducted two-sided Mann–Whitney U test on the results of the population genomics analyses by using SciPy library v1.11^25^.

### Supplementary Information

Supplementary Information.

## Data Availability

Whole genome resequencing data have been deposited at the NCBI Sequence Read Archive and are publicly available under accession number PRJNA1101173 (BioProject) and SRR28712676 to SRR28712698 (SRA).
